# Early Direct Antiglobulin Test Negativity after Bendamustine and Rituximab Treatment in Chronic Lymphocytic Leukemia: Two Cases

**DOI:** 10.4274/tjh.2017.0464

**Published:** 2018-11-13

**Authors:** Rafet Eren, Elif Suyanı

**Affiliations:** 1 University of Health Sciences, İstanbul Training and Research Hospital, Clinic of Hematology, İstanbul, Turkey

**Keywords:** Chronic lymphocytic leukemia, Autoimmune hemolytic anemia, Bendamustine, Rituximab

## To the Editor,

Autoimmune hemolytic anemia (AIHA) can emerge at any stage of chronic lymphocytic leukemia (CLL); furthermore, patients can present with AIHA before diagnosis [1]. Although direct antiglobulin test (DAT) positivity is one of the hallmarks of AIHA, it was also demonstrated to be associated with advanced disease [2] and poor prognosis [3] independent of hemolytic anemia in CLL patients [3]. Here we present two CLL patients with AIHA whose DAT results became negative shortly after receiving bendamustine-rituximab (BR) chemotherapy.

## Case 1

A 69-year-old male patient who was being followed without treatment for CLL in Rai stage 2 for 6 months presented with abdominal pain and jaundice. Laboratory tests were as follows: leukocytes: 55,140/µL, lymphocytes: 51,240/µL, hemoglobin: 5.3 g/dL, platelets: 46,000/µL, indirect bilirubin: 2.89 mg/dL, haptoglobin: 2 mg/dL, lactate dehydrogenase (LDH): 1585 U/L, and DAT positive for Immunoglobulin G (IgG) (no titer provided). Imaging studies showed compressing conglomerate lymph node masses in the abdomen. The patient was started on steroid and BR treatments. The hemoglobin value rose to normal levels and DAT became negative after 3 cycles of BR. The patient received 6 cycles of BR chemotherapy and steroids were interrupted at the 5^th^ month of treatment. The patient has been followed in remission for 1 year.

## Case 2

A 75-year-old female patient who was being followed without treatment with the diagnosis of CLL in Rai 0 stage for 8 years was admitted due to weakness and fatigue. Laboratory tests were as follows: leukocytes: 78,840/µL, lymphocytes: 67,020/µL, hemoglobin: 6.3 g/dL, platelets: 255,000/µL, indirect bilirubin: 2.58 mg/dL, LDH: 504 U/L, haptoglobin: 1 mg/dL, corrected reticulocyte count: 5.2%, and DAT positive for IgG (4+). The patient was started on steroid treatment and subsequently BR therapy was added due to increased lymphocyte doubling time. After the first cycle, the DAT titer dropped to 3+. Hemoglobin value rose to normal levels and DAT became negative after 3 cycles of BR. Steroids were ceased at the 7^th^ month of treatment; The patient completed 6 cycles of BR and has been followed in remission for 1 year.

While the standard approach in CLL patients with AIHA is steroids, systemic chemotherapy is recommended in refractory cases and in patients requiring treatment for CLL [1]. Although first-line therapy in CLL patients is the fludarabine-cyclophosphamide-rituximab regimen, the wide use of BR chemotherapy, especially in advanced-age patients, has brought up the application of this combination in patients with AIHA [4,5]. In a recent study including 26 CLL patients who had AIHA and received BR, the response rate was 81% for AIHA and 77% for CLL [4]. Similarly, our patients also responded well in terms of CLL and AIHA. The most striking point was that DAT became negative in a short period of time (after 3 cycles of BR).

In conclusion, in addition to being a plausible option in advanced-age CLL patients, BR seems to be an important treatment of choice in terms of eliminating the poor prognostic factor of DAT positivity and assuring safe cessation of steroid treatment due to rapid achievement of DAT negativity.
